# Fibrotic Pulmonary Sarcoidosis—From Pathogenesis to Management

**DOI:** 10.3390/jcm14072381

**Published:** 2025-03-30

**Authors:** Ewa Łyżwa, Jacek Wakuliński, Monika Szturmowicz, Witold Tomkowski, Małgorzata Sobiecka

**Affiliations:** 1 1st Department of Lung Diseases, National Research Institute of Tuberculosis and Lung Diseases, 01-138 Warsaw, Poland; monika.szturmowicz@gmail.com (M.S.); w.tomkowski@igichp.edu.pl (W.T.); m.sobiecka@igichp.edu.pl (M.S.); 2 Department of Radiology, National Research Institute of Tuberculosis and Lung Diseases, 01-138 Warsaw, Poland; j.wakulinski@igichp.edu.pl

**Keywords:** sarcoidosis, pulmonary fibrosis, interstitial lung disease, HRCT pattern, molecular mechanisms, complications

## Abstract

Sarcoidosis is a multiorgan, granulomatous disease of an unknown etiology. The characteristic feature of the disease is the formation of noncaseating granulomas. Spontaneous resolution occurs in most patients, but the clinical course may be chronic or progressive, complicated by pulmonary fibrosis, which is a major cause of mortality in sarcoidosis. Recent studies have provided new information on the immunological mechanisms of pulmonary fibrosis. Its pathogenesis includes the alteration of lymphocyte activity and the imbalance between their subpopulations, the polarization of macrophages to the profibrotic phenotype, and an imbalance between the activity of metalloproteinases and their tissue inhibitors. A multidisciplinary approach is required for the optimal management of fibrotic pulmonary sarcoidosis. Clinical symptoms, serum biomarkers, imaging, pulmonary function test results, other organ involvement, comorbidities, and complications should be considered when assessing disease activity and selecting the most appropriate treatment. The use of anti-inflammatory drugs is often discussed. There has been no consensus reached on whether antifibrotic agents should be added or used in monotherapy as initial treatment in such cases. This article will review all the information on fibrotic pulmonary sarcoidosis and present factors associated with fibrosis development, prognosis, and treatment options.

## 1. Introduction

Sarcoidosis is a multiorgan, granulomatous disease of unknown etiology. It is thought to be an immunological response to some undefined antigens in genetically predisposed patients. The characteristic feature of the disease is the formation of noncaseating granulomas in place of an inflammatory process. The lungs are the most commonly affected organ, with granulomas occurring primarily along lymphatic tracks in interlobular septa and broncho-vascular bundles [1]. The course of sarcoidosis is variable, with some patients being asymptomatic and others presenting with both generalized and organ-specific symptoms. The spontaneous resolution occurs in 60–80% of patients, but in 20–40% of cases, the clinical course is chronic or progressive, complicated by organ failure or fibrosis. A significant challenge is the lack of clearly defined predictors of the development of interstitial lung fibrosis in the course of sarcoidosis. Moreover, pulmonary fibrosis may also be the first manifestation of the disease. Genetic polymorphisms, alteration of lymphocyte and macrophage activity, and environmental conditions are key factors leading to the development of fibrosis. This article presents current knowledge regarding pathogenesis, clinical and radiological manifestations, and management of fibrotic pulmonary sarcoidosis (fPS).

## 2. Methods

An online search was performed up to 09/2024 using PubMed search engines. Two authors (EŁ and MS) performed the search and jointly selected articles for inclusion. All types of articles were included. Keywords for the searches included primarily “sarcoidosis”, “fibrotic pulmonary sarcoidosis”, “pulmonary fibrosis”, “HRCT pattern in sarcoidosis”, and “molecular mechanisms in sarcoidosis”.

## 3. Pathogenesis

### 3.1. Genetic Background of Fibrotic Pulmonary Sarcoidosis

The genetic background of sarcoidosis seems undeniable. Immunological and environmental triggers that may induce the disease development and its specific course in genetically predisposed individuals remain an area of investigation. Diverse epidemiology may suggest genetic differences among ethnicities [2]. A higher incidence of sarcoidosis has been reported in Northern European countries and African Americans in the United States [3,4]. The incidence and age of onset of sarcoidosis also vary between genders [5,6]. The occurrence of familial sarcoidosis has been confirmed. Numerous studies have shown an increased risk of sarcoidosis among first and second-degree relatives of sarcoidosis patients compared to controls [7,8,9,10,11,12].

All phagocytized antigens are presented to T lymphocytes by molecules belonging to the major histocompatibility complex (MHC). The genes encoding proteins of the human histocompatibility complex (HLA) show a large polymorphism associated with different sarcoidosis susceptibilities, phenotypes, and organ involvement [2,13,14]. HLA polymorphism may provide a background for the development of sarcoidosis. However, no specific HLA associated with the development of pulmonary fibrosis has been identified. In addition, other genes may play a role in the development and course of the disease. Significant polymorphism differences in GREM1 (gremlin 1) were found between sarcoidosis patients with and without chest radiographic abnormalities. Patients with the GREM1 CC genotype at position rs1919364 were more than 6 times more likely to develop pulmonary fibrosis [15]. Levin et al. found that less common variants of ANXA11 (annexin 11), the gene associated with cell apoptosis and proliferation, were independently associated with disease susceptibility and advanced radiographic stage [16]. Transforming growth factor (TGF)-β, responsible for repair processes, plays a critical role in lung fibrosis. Kruit et al. have suggested a role for TGF-β 3 genetic variation in the predisposition to pulmonary fibrosis [17]. Toll-like receptor (TLR) 3 polymorphism, Leu412Phe (TLR3 Leu412Phe, L412F; rs3775291) variant, which results in accelerated disease progression and increased risk of mortality in idiopathic pulmonary fibrosis (IPF), was also found to be significantly associated with the development of a chronic clinical phenotype in pulmonary sarcoidosis [18,19]. In contrast to IPF, the mucin 5b polymorphism has no association with fPS [20]. Studies on genetic polymorphisms in fPS are limited. However, advanced technologies such as next-generation sequencing (NGS) and whole-exome sequencing (WES) may shed new light on the genetic background of sarcoidosis and pulmonary fibrosis in their course [3].

### 3.2. Molecular Mechanisms

The pathogenesis of pulmonary fibrosis in sarcoidosis includes alteration of lymphocyte activity in favor of the T helper 2 (Th2) subpopulation, the polarization of macrophages to the profibrotic M2 phenotype, an imbalance between the population of Th17 and T regulatory (Treg) lymphocytes, and between the activity of metalloproteinases and their tissue inhibitors. Aware of many immunological mechanisms and signaling pathways involved in pulmonary fibrosis in sarcoidosis, and the impossibility of describing all of them, we will focus on the abovementioned issues in the following chapters.

#### 3.2.1. Th1/Th2 Lymphocytes

T CD4 lymphocytes remain key players in the pathogenesis of sarcoidosis. The CD4/CD8 ratio in bronchoalveolar lavage fluid (BALF) from sarcoid patients is elevated compared to healthy controls, and a significant decrease in the CD4/CD8 ratio is observed along with the radiographic stage of sarcoidosis [21,22]. Lymphocyte T CD4 subpopulations, distinguished by their cytokine profiles, have been named T helper (Th) 1, Th2, and Th17. The characteristic cytokines of Th1 lymphocytes are interferon (IFN)-γ and interleukin (IL)-2. Th1 lymphocytes, acting through IFN-γ and CD40 ligand, enhance the ability of the classically activated, proinflammatory subtype of macrophages, M1, to kill microorganisms [23,24,25]. As high levels of IFN-γ, IL-2, tumor necrosis factor (TNF)-α, and high T-cell expression of chemokine receptors CXCR3 and CCR5 were found in BALF, sarcoidosis was initially considered a Th1-driven disease [26,27]. Recently, however, the involvement of Th2 in the pathogenesis of lung fibrosis has been postulated [28,29]. IL-4, IL-5, and IL-13 secreted by Th2 are mediators of tissue fibrosis. IL-13 stimulates fibroblast proliferation and collagen production and may also stimulate the production and activation of TGF-β [29]. Studies on the Th1/Th2 switch in sarcoidosis are limited. Abedini et al. found elevated serum levels of IL-4 and IL-13 in patients with sarcoidosis compared to healthy controls and patients with tuberculosis, but information on sarcoidosis stages was not available [30]. Hauber et al. also showed elevated IL-13 levels in the blood and BALF of sarcoid patients; however, IL-13 mRNA expression was significantly increased in the stage I group compared to the stage II/III group, and no stage IV patients were included. Importantly, the authors suggested that alveolar macrophages, rather than lymphocytes, were an important source of IL-13 [31]. Ten Berge et al. demonstrated a significantly higher proportion of IL-4-producing cells in peripheral blood samples of sarcoid patients compared to controls; however, only individuals with stages I and II of sarcoidosis were included in this study [32]. Patterson et al. investigated the panel of circulating cytokines in patients with chronic sarcoidosis and showed a significantly decreased IL-5 level in non-fibrotic compared to fibrotic sarcoidosis patients and controls. The suppression of Th2 cells in the circulating immune system of sarcoidosis patients was suggested in this study [33]. As shown above, the Th1/Th2 switch remains an area of investigation. Further studies involving patients with all stages of sarcoidosis are needed.

#### 3.2.2. Macrophages

Recently, the polarization of M1 and M2 macrophages has been extensively studied in sarcoidosis. M1 macrophages are induced by lipopolysaccharide (LPS) and cytokines secreted by Th1 lymphocytes. They produce proinflammatory substances, mainly IFN-γ, IL-12, and TNF-α. The polarization of M2 macrophages depends on IL-4 and IL-13 released by Th2 lymphocytes, immune complexes, and glucocorticoids [23,24,34]. Nevertheless, M1 and M2 macrophage categorization may not be completely adequate to reflect macrophage heterogeneity. Moreover, macrophage diversity in vivo is more complicated than in vitro because macrophages are exposed to different stimuli and their phenotype depends on tissue region and disease phase [34]. Several molecules are used to detect macrophage patterns. CC chemokine ligand 18 (CCL18), secreted by alternatively activated macrophages, has been implicated in the pathogenesis of fibrotic lung diseases and indicates a worse prognosis in IPF [35,36]. Wiken et al. showed increased gene expression of CCL18 in sarcoid patients compared to controls, regardless of disease stage. However, the study group included only one patient with stage IV sarcoidosis. There was no difference between the other stages. Interestingly, IL-10 and CCR2 used as markers of M2 macrophages did not indicate M2 polarization [37]. Prasse et al. showed that BAL-derived cells from patients with stage IV sarcoidosis produced significantly higher levels of CCL18 than those from patients with stages I-III. Alveolar and interstitial macrophages were the main source of this chemokine. IL-4, IL-13, and IL-10 significantly up-regulated CCL18 gene expression and protein production. Lung fibroblasts significantly enhanced CCL18 production by macrophages [36]. In a study evaluating the polarization of macrophages in granulomas in pulmonary sarcoidosis, Shamaei et al. used CD163 as a marker for M2 and found a shift toward M2 macrophages in chronic cases of pulmonary sarcoidosis. Importantly, a significant correlation was observed between the radiological stage and CD163 expression score [38]. Wojtan et al. retrospectively analyzed BALF from patients with a wide spectrum of interstitial lung diseases (ILDs), focusing on CD40 as a marker for M1 and CD163 as a marker for M2 macrophages. When comparing the groups, they found no significant difference in macrophage patterns. Only sarcoid stage I-III patients were included [39]. It remains controversial whether CD163 can be a reliable M2 marker when used alone [24]. Further studies on macrophage polarization, including sarcoid patients with pulmonary fibrosis, are needed.

#### 3.2.3. Th17, Th17.1, Treg Lymphocytes

Th17 lymphocyte differentiation in response to extracellular bacterial and fungal infections and in autoimmune diseases such as rheumatoid arthritis, Sjögren’s syndrome, and allergic lung diseases can be induced under the proinflammatory cytokines IL-1, IL-6, and IL-23 [40,41]. The Th17 subpopulation produces mainly IL-17 and IL-22 and is determined by the transcription factor retinoic acid-related orphan receptor γt (RORγt). The combined action of IFN-γ and IL-12 can induce Th17 cells to develop into Th17.1 cells. The co-expression of the IFN-γ and IL-17, and the transcription factors T-bet and RORγt (T-bet+ RORγt+) characterize Th17.1 cells. Such lymphocytes combine the pro-inflammatory capabilities of both Th17 and Th1 lymphocytes [42]. Kaiser et al. found that the percentage of T-bet+ RORγt+ T cells was significantly higher in the BALF from patients with “nonchronic” sarcoidosis (defined as the absence of pathological changes and clinical symptoms within 2 years of disease onset) than with chronic disease course [43]. In contrast, Broos et al. found significantly higher BALF Th17.1 proportions at the time of diagnosis in patients who developed progressive chronic sarcoidosis requiring treatment compared to patients who resolved within 2 years of follow-up [44]. Ten Berge et al. examined peripheral blood, BALF, and lung mucosal biopsies of newly diagnosed sarcoidosis patients (stage I and II only) and found increased proportions of IL-17A+ memory cells, especially IL-17A and IFN-γ double-producing cells in the blood and BALF. IL-17A-expressing cells were most commonly found in areas of granuloma formation in histopathological material [32]. Recently, Jiang et al. in a study on mice have demonstrated the progression of sarcoidosis from granuloma formation to pulmonary fibrosis. No granulomas were found in the IL-17A knockout mice group, whereas granulomatous inflammation in the other group was observed. Pulmonary fibrosis was also seen only in the second group. IFN-γ and TGF-β were released in both groups during the observation period. The authors concluded that without IL-17A, upregulation of TGF-β and IFN-γ could not induce lung fibrosis [45]. Zhang et al. investigated the Th17.1 dominant subpopulation of CD4 lymphocytes in the BALF of sarcoid patients. The percentage of Th17.1 cells was significantly higher compared to the IPF patients included in this study. Furthermore, patients with fPS had a higher proportion of circulating Tregs than those with non-fibrotic stages [46]. In line with Zhang et al., other authors have also found a significantly increased proportion of circulating Tregs in patients who develop chronic disease, demonstrating at the same time reduced functional capacity [47,48,49]. Broos et al. found an impaired survival of circulating Tregs, associated with increased apoptotic susceptibility in patients with pulmonary sarcoidosis. According to the authors, the number of circulating Tregs at diagnosis could be a biomarker indicating the need for immunosuppressive therapy [47]. In contrast, Treg cells in sarcoid lesions were found to secrete pro-inflammatory cytokines that are likely to promote a chronic inflammatory process in the affected lung [48]. Although Th17.1 and Tregs lymphocyte subpopulations have been studied for a long time, recent studies shed new light on this issue, and further evaluation is needed.

**Figure 1:**
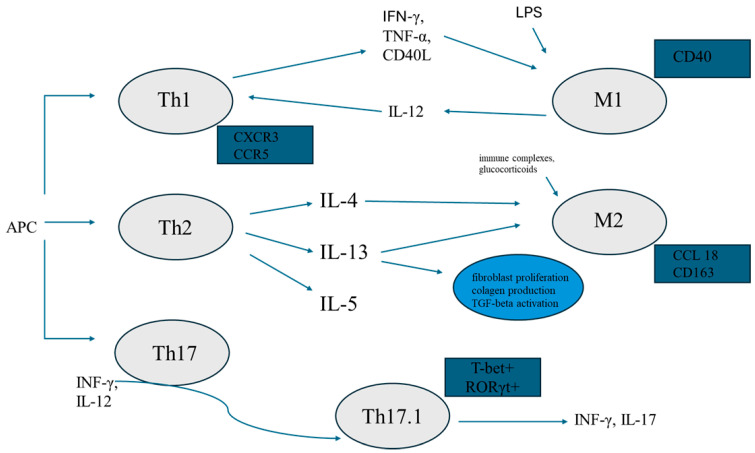
Graphic summary of the interaction between Th lymphocyte and macrophages. APC—antigen-presenting cell; Th1, 2, 17, 17.1—T helper 1, 2, 17, 17.1 lymphocytes respectively; IFN-γ—interferon-γ; TNF-α—tumor necrosis factor-α; LPS—lipopolysaccharide; IL—interleukin; M1—pro-inflammatory subtype of macrophages; M2—alternatively activated macrophages; TGF-β—transforming growth factor-β; CCL18—CC chemokine ligand 18.

The abovementioned interaction between Th lymphocytes and macrophages is summarised in Figure 1.

## 4. Clinical Features

The clinical presentation and progression of fPS are characterized by significant variability among affected patients, ranging from asymptomatic and sparse disease to inevitable progressive pulmonary fibrosis, despite treatment, and ultimately to death. In patients with fPS, lung fibrosis is diagnosed during long-term follow-up in about two-thirds of patients, while it is the first manifestation of the disease in the remaining patients [50].

Pulmonary fibrosis, referred to as stage IV on chest radiography, affects approximately 2–5% of patients with sarcoidosis at presentation. More common is progression to higher stages, usually after 5 years or more of follow-up: from stage I to stage III or IV in 0–26% of cases, from stage II to stage IV in 8–16% of cases, and from stage III to stage IV in 29–41% of cases [51].

The most common complaints are dyspnea (80%), chronic cough (51.4%), and sputum production (18.3%) [52]. The mechanism of dyspnea is complex and can result from lung function impairment, muscle weakness, and pulmonary hypertension (PH) [53,54].

A rare symptom but indicative of the presence of complications in patients with fPS is hemoptysis, which occurs in about 3% of patients. This is most commonly the result of chronic pulmonary aspergillosis (CPA), but may also be associated with other complications such as mycobacterial infection, pneumonia, or pulmonary embolism [52,55,56].

Fatigue is also a symptom of sarcoidosis, which can result from chronic inflammation, impaired lung function, muscle involvement, and PH [57]. Surprisingly, no correlation between disease stage and Fatigue Assessment Scale (FAS) score has been observed. Furthermore, it is also reported in patients with disease remission [58].

In patients with fPS, the physical examination is often normal, despite extensive lung fibrosis. On auscultation, some patients have crackles (28%) due to bronchiectasis and advanced pulmonary fibrosis, and wheezing (6%) due to bronchial distortion from airway-centered fibrosis and airway obstruction [52,59]. However, auscultation findings may not always reflect the advancement of interstitial and fibrosing changes. Finger clubbing is quite uncommon.

## 5. Radiological Aspects of Fibrotic Pulmonary Sarcoidosis

**Figure 2:**
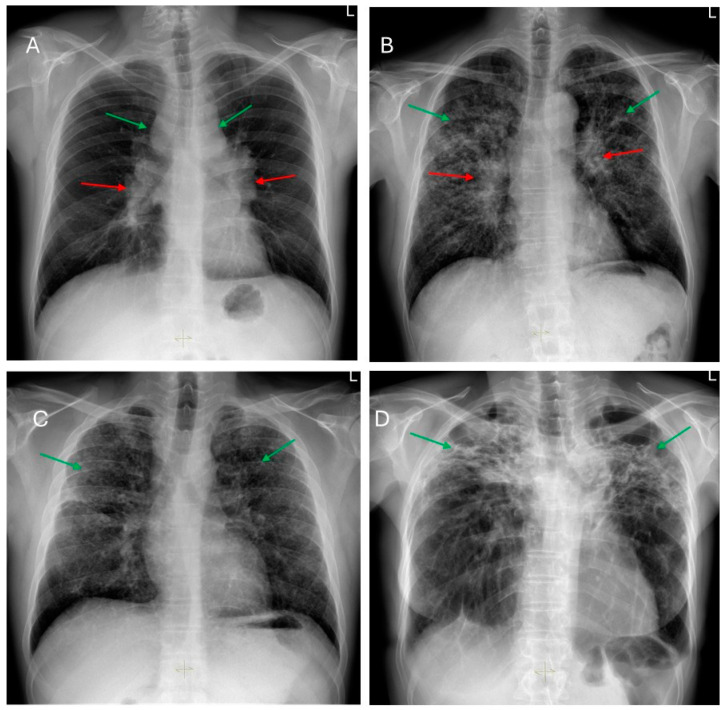
Frontal chest radiographs of patients with: (**A**)—Stage I—bilateral hilar (red arrows) and paratracheal (green arrows) lymph node enlargement. (**B**)—Stage II—diffused, multiple small nodular opacities predominantly located in the upper lung lobes (green arrows) and bilateral symmetric hilar lymph node enlargement (red arrows). (**C**)—Stage III—diffused, multiple small nodular opacities predominantly located in the upper lung lobes (green arrows). (**D**)—Stage IV—prominent upper zones fibrosis and volume loss (green arrows).

Radiological imaging is a critical tool, guiding the initial diagnosis, ongoing evaluation, prognostication, and therapeutic decision-making. Historically, the classification of thoracic sarcoidosis relied heavily on the chest radiograph, with the seminal staging system described by Karl Wurm and later revised by Guy Scadding in 1961. Based on radiographic findings such as hilar lymphadenopathy and parenchymal involvement, this system stratifies sarcoidosis into five stages (0–IV) [60]. Figure 2 shows their main features. Stage IV denotes fibrotic disease and is defined by architectural distortion, volume loss, and advanced scarring, typically most pronounced in the upper lobes [60].

However, although universally available and cost-effective, chest radiographs lack the sensitivity necessary to precisely capture early fibrotic changes or differentiate between active granulomatous inflammation and established fibrosis. The advent of high-resolution computed tomography (HRCT) revolutionized the imaging landscape, enabling clinicians and researchers to identify subtle abnormalities earlier than with traditional radiography, differentiate pathologic patterns, and quantify the extent of fibrotic changes [61,62]. HRCT findings alone or combined with non-imaging markers facilitate more accurate prognostication and guide therapeutic interventions earlier in the disease course [63,64,65,66]. HRCT improves the reproducibility and reliability of imaging evaluations, enabling better comparisons across centers and clinical trials [62,67,68,69].

Even though there have been propositions to shift staging from chest X-ray to HRCT, current BTS guidelines recommend that Scadding’s staging system remain based solely on chest radiographs [62,70]. HRCT’s role is to supplement the diagnostic process by providing detailed insights into the extent and nature of fibrotic changes, rather than redefining staging criteria. This distinction ensures consistency in diagnosis and classification while utilizing HRCT for enhanced evaluation.

### 5.1. Key HRCT Findings in fPS

Several characteristic HRCT patterns have been identified in fPS, each with distinct clinical correlations and prognostic implications. These include micronodules, ground-glass opacities, bronchial distortion, honeycombing, linear fibrosis, and the formation of cysts or bullae [61,71,72,73,74].

The radiologic manifestations of fPS evolve over time. Early fibrotic changes may manifest as subtle reticular opacities and small areas of ground-glass attenuation. As the disease advances, progressive volume loss, pulmonary fissure distortion, and major bronchi displacement reflect ongoing architectural remodeling. Over time, conglomerate fibrosis and parahilar scarring emerge, often resulting in traction bronchiectasis and marked distortion of central airways. In the late stages, honeycombing and peripheral bullae dominate, culminating in severe functional impairment, chronic respiratory failure, and increased vulnerability to complications such as pneumothorax and aspergillosis [56,66,72,74,75].

#### 5.1.1. Micronodules and Ground-Glass Opacities (GGOs)

Micronodules, typically arranged in a perilymphatic distribution and predominantly affecting the upper and middle lung zones, are among the most common radiological findings in sarcoidosis [74]. They suggest active granulomatous inflammation and are seen in up to 70–90% of patients. Yet, in fPS, their prevalence decreases substantially as the disease shifts towards an irreversible scarring process. GGOs may coexist with micronodules and similarly imply ongoing inflammatory activity. Although GGOs can be reversible with therapy, their presence in fibrotic disease may reflect a residual inflammatory component superimposed on fibrosis. Recent large-scale data demonstrate that while GGOs are associated with worse pulmonary function, they do not independently predict reduced survival, underscoring the complexity of correlating functional and prognostic outcomes with a single imaging feature [66].

#### 5.1.2. Bronchial Distortion

**Figure 3:**
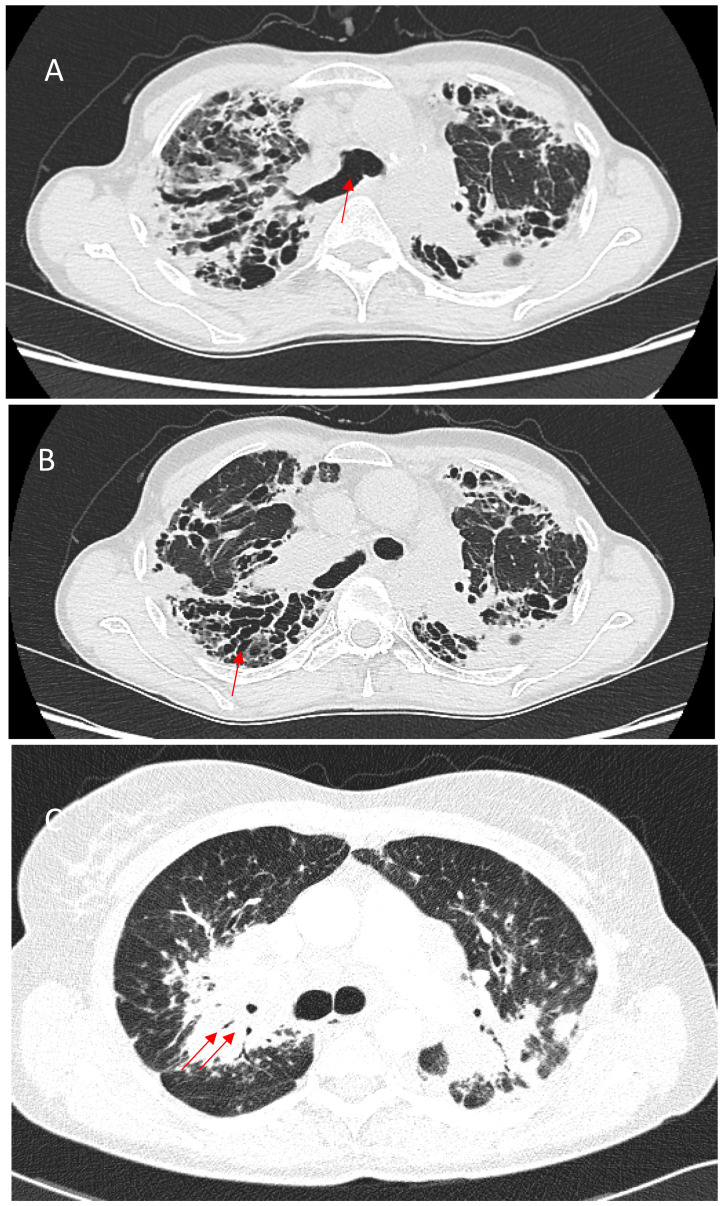
(**A**)—Right upper lobe fibrosis predominantly located in the posterior aspect of the lobe, causing retraction of the right upper lobe bronchus (red arrow). (**B**)—Marked traction bronchiectasis (red arrow). (**C**)—Bronchial narrowing caused by perihilar fibrotic masses (red arrows).

Bronchial distortion is considered one of the hallmark patterns in fPS, identified in nearly half of such cases [73,75]. It reflects airway involvement by granulomatous infiltration in and around the airways, bronchial angulation, and traction bronchiectasis due to fibrotic pulmonary changes [59]. Luminal narrowing, the thickening of broncho-vascular bundles, sometimes accompanied by conglomerate parahilar fibrotic masses exceeding 3 cm, can be observed. Over time, the progressive shrinkage of these fibrotic conglomerates can exacerbate bronchial deformation, impairing airflow and contributing to obstructive ventilatory defects. Though important for characterizing fibrotic involvement, bronchial distortion is not as strongly linked to mortality as some other HRCT patterns [66]. Bronchial sarcoidosis with multiple segmental or lobar stenosis may mimic the clinical picture of chronic obstructive pulmonary disease, and it can cause atelectasis on imaging, requiring differentiation from pulmonary neoplasm [76]. Verleden et al. identified granulomatous remodeling as a reason for small airway loss using a combination of CT scanning, micro-CT scanning, and histological examination of seven explant lungs with end-stage fPS [77]. Figure 3 shows some of the abovementioned bronchial distortions.

#### 5.1.3. Honeycombing

**Figure 4:**
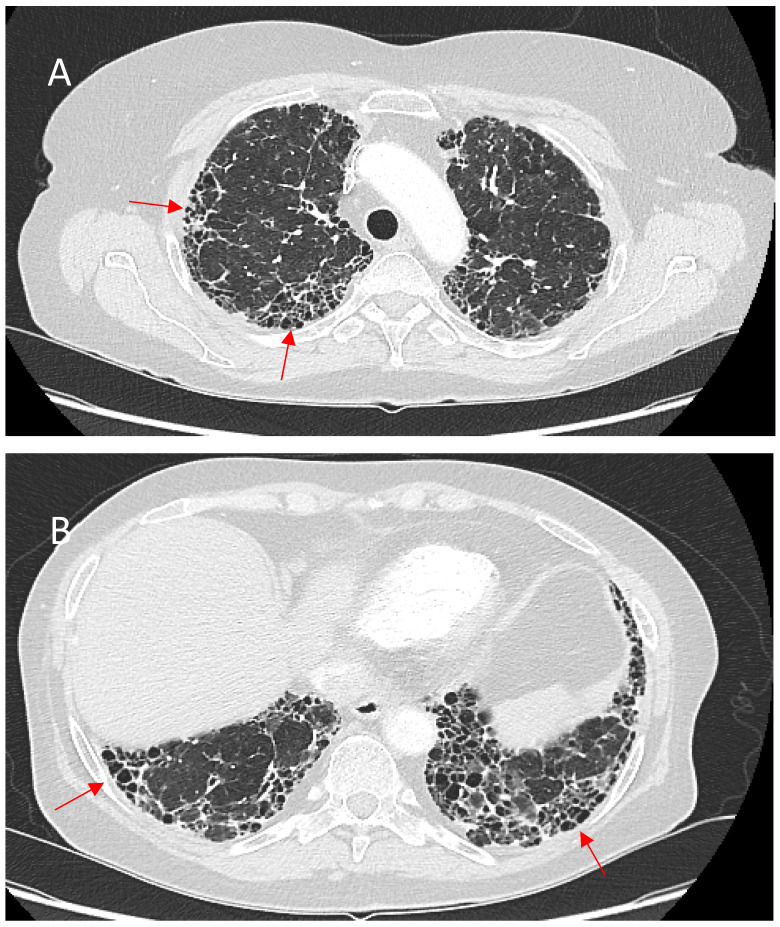
(**A**)—Typical manifestation of honeycombing (red arrows) in fibrotic sarcoidosis affecting the periphery of the upper lobes. (**B**)—Atypical manifestation of honeycombing (red arrows) in fibrotic sarcoidosis affecting the periphery of the lower lobes mimicking UIP pattern.

Honeycombing, defined by clustered cystic air spaces with thick fibrous walls, is a critical imaging manifestation in fPS. Unlike IPF, where honeycombing is characteristically basal and peripheral, sarcoidosis-associated honeycombing often involves the upper and perihilar lung zones. When this upper zone pattern predominates, it can serve as a distinguishing radiographic feature differentiating sarcoidosis from IPF. However, fPS may occasionally present with the usual interstitial pneumonia (UIP)-like pattern, including basal subpleural honeycombing. This less typical distribution can portend a more severe course [78]. Emerging evidence indicates that basal subpleural honeycombing in sarcoidosis, resembling the pattern seen in IPF, strongly correlates with worse survival. In a large cohort study of 240 patients with fPS, basal subpleural honeycombing, along with extensive (>20%) fibrotic involvement, was associated with significantly reduced survival and more severe impairment in pulmonary function [66]. Figure 4 shows the abovementioned typical and atypical manifestations of honeycombing in fPS.

#### 5.1.4. Linear Fibrosis

**Figure 5:**
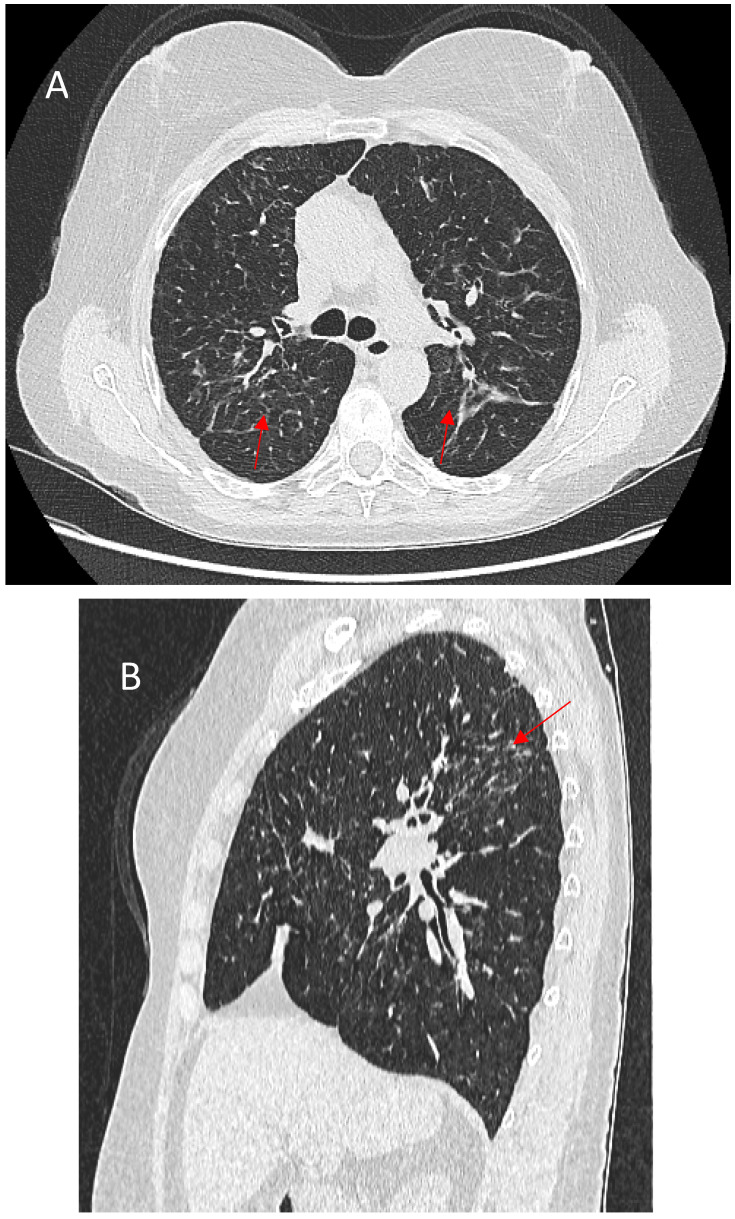
(**A**)—Typical appearance of linear fibrosis (red arrows) in sarcoidosis affecting the upper and posterior parts of the lungs. (**B**)—Sagittal reconstruction depicting the greatest architectural distortion and volume loss (red arrow) in the posterior part of the upper lung zones.

Linear fibrotic changes, including irregular reticulation, thickened interlobular septa, and hilar peripheral lines, occur in roughly one-quarter of fPS cases. Although typically less functionally debilitating than honeycombing or extensive bronchial distortion, linear fibrosis is often irreversible and may contribute to mild restrictive abnormalities. Its prognostic implications are more modest, and it generally does not portend as severe outcomes as other fibrotic patterns [66]. Figure 5 shows linear fibrotic changes and architectural distortion.

#### 5.1.5. Bullae and Cysts

**Figure 6:**
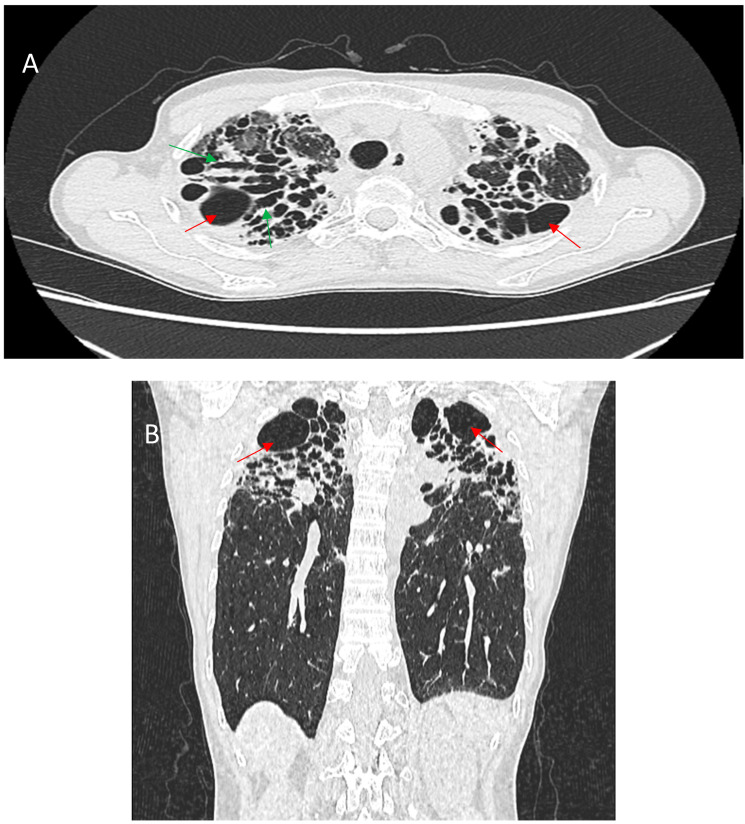
Enhanced axial CT scan (**A**) and coronal reconstruction (**B**) show a prominent typical pattern of fibrosis. There is severe upper zone volume loss associated with architectural distortion, cystic spaces (red arrows), and traction bronchiectasis (green arrows).

In advanced fPS, peripheral cysts or bullae can form due to a check-valve mechanism induced by fibrotic changes. The presence of large bullae has been linked to reduced lung transfer factor for carbon monoxide (TLCO) and worse survival [66]. Pneumothorax may be a complication of bullous disease or fibrosis late in the course of sarcoidosis [52,79]. Figure 6 shows cystic spaces, volume loss, and traction bronchiectasis included in a typical pattern of fibrosis.

### 5.2. The Role of FDG-PET/CT in Assessing Disease Activity

Positron emission tomography with 2-deoxy-2-[18F]fluoro-D-glucose integrated with computed tomography (FDG-PET/CT) provides metabolic and structural information that can complement HRCT findings by identifying sites of active granulomatous inflammation. Granulomas characteristically demonstrate increased FDG uptake, distinguishing potentially reversible inflammatory lesions from low-uptake fibrotic masses [80]. However, FDG-PET/CT has certain limitations. The absence of standardized SUVmax thresholds for defining active disease complicates its use in routine clinical practice. Although some studies have shown that decreases in SUVmax after therapy predict lower relapse rates, robust prospective trials are needed to establish validated cutoffs and integrate FDG-PET/CT findings into treatment algorithms [81,82,83].

### 5.3. Future Directions in Imaging

Recent analyses have described additional radiological findings, including central-peripheral (C-P) bands that connect central and subpleural consolidations along lymphatic drainage routes, and pleuroparenchymal fibroelastosis (PPFE)-like lesions [84]. These emerging patterns highlight the morphological heterogeneity of fPS and may reflect distinct pathophysiological pathways. As more patients undergo HRCT as part of routine evaluation, previously underrecognized patterns may gain prognostic and therapeutic relevance.

The importance of reproducibility and standardization is increasingly acknowledged. Interreader variability in diagnosing and quantifying fibrosis is a recognized challenge, potentially confounding clinical decision-making and research outcomes. To address this, automated image analysis tools and machine-learning algorithms are being investigated for their ability to quantify fibrotic burden and predict outcomes more consistently than subjective assessments alone. Further validation of these approaches may improve prognostication, support multi-center trials, and guide personalized therapy.

Although HRCT and PET/CT have enhanced our understanding of fPS, factors that determine the transition from granulomatous inflammation to irreversible fibrosis remain unknown.

## 6. Pulmonary Function Tests

TLCO reduction is the most common abnormality among patients with fPS [52,85]. Normal TLCO is a good predictor of the absence of severe gas exchange impairment. However, it has been reported to be a less sensitive index than oxygen desaturation during a six-minute walk test (6MWT) in assessing gas exchange impairment in sarcoidosis [86]. Lower TLCO is associated with a higher risk of clinical worsening events in sarcoid patients with extensive lung fibrosis on HRCT [87]. Moreover, TLCO is an independent predictor of mortality. [88] Higher TLCO impairment was observed in stage IV sarcoid patients with PH compared to patients without PH [54].

Restrictive lung ventilation patterns of functional disorders have been observed more frequently than obstructive ventilatory defects [1,52,59,89]. The correlation of pulmonary function test (PFT) results with computed tomography (CT) patterns suggests that ventilatory restriction and low diffusion capacity correlate with honeycombing [73]. Airflow obstruction in fPS may be caused by airway distortions, bullous changes, and peribronchial or peribronchiolar fibrosis [59,73]. Kouranos et al. have found that mixed ventilatory defects were more common than restrictive and obstructive disorders in patients with stage IV sarcoidosis. Furthermore, in this study, mortality was associated with mixed and restrictive ventilatory defects compared to patients with obstructive disorders [88]. Significant differences in pulmonary function phenotypes by race and sex were noted, with blacks more likely to have restrictive and whites more likely to have obstructive lung function defects. Fibrocystic disease was more common in patients with restrictive or mixed ventilatory defects in this study [90].

Pulmonary function tests are also a very important part of monitoring the course of the disease and assessing its progression. In some patients with fPS, PFT results remain stable or improve during observation, partly due to the therapy applied [52,85,91]. Nevertheless, progressive fibrosing interstitial lung disease (PF-ILD) phenotype, defined as (a) a relative decline in the forced vital capacity (FVC) of at least 10% of the predicted value, (b) a relative decline in the FVC of 5% to less than 10% of the predicted value, and worsening of respiratory symptoms or an increased extent of fibrosis on high-resolution CT, or (c) worsening of respiratory symptoms and an increased extent of fibrosis within 24 months, can be observed in some patients with fPS [92]. Schimmelpennink et al., in a study including 106 patients with advanced pulmonary sarcoidosis and severely impaired TLCO who had at least 10% fibrosis on CT, found PF-ILD in 15% of patients, and it was one of the independent predictors of mortality and lung transplantation. It has been suggested that without such a low TLCO criterion, the percentage of patients fulfilling PF-ILD criteria would have been significantly lower [85]. Without the CT criterion, the percentage might have been higher, as shown in another study [93]. Hambly et al. found PF-ILD in 29 of 92 patients (31%) with sarcoidosis compared to 42–49% of patients with the majority of connective tissue disease (CTD)-ILD group; 25–37% of patients with Sjögren’s syndrome and systemic lupus erythematosus, and 41–56% of patients with idiopathic non-specific interstitial pneumonia (NSIP), occupational ILD and smoking-related ILD [94]. Some authors point out that PFT worsening in sarcoidosis occurs less frequently than in other fibrotic ILD and that mainly radiological progression should draw attention during, e.g., treatment qualification [95].

The simple, repeatable, and inexpensive 6-MWT is another method used to assess functional lung capacity. This test is most commonly used in the initial assessment of patients to monitor disease progression and treatment response and as a secondary endpoint in clinical trials evaluating the efficacy of new therapies for pulmonary sarcoidosis. Patients with fPS tended to have shorter walking distances, greater desaturation, and higher heart rates [89,96]. However, some studies have found no difference in the walking distance among patients with sarcoidosis stages I to IV [86]. Higher degrees of desaturation correlate with PH in sarcoidosis [97]. In another study, the walking distance was the most significant determinant of survival in a large group of patients with sarcoidosis-associated PH at a cut-off of 300 m [98]. Lettieri et al. proposed the use of a new index, the distance-saturation product (DSP), that combined both the distance walked and the lowest oxygen saturation (SpO2). For example, a walk distance of 300 m on the 6MWT with SpO2 falling to 90% would have a DSP of 270 m% (e.g., 300 × 0.90). This parameter was a good predictor of survival in IPF patients [99]. Alhamad et al. performed an interesting study to investigate the relationship between the 6MWT, physiological parameters, and CT patterns in a large group of patients with sarcoidosis in which more than 40% had evidence of fibrosis on CT. Although patients with pulmonary fibrosis tended to achieve shorter distances than those without fibrotic changes, the difference was not statistically significant. However, the differences in DSP values were significant. The authors suggested that DSP could help to identify a subpopulation of patients with pulmonary fibrosis [100]. In general, exercise intolerance in patients with sarcoidosis may be associated with other factors, such as chronic fatigue, cardiac involvement, and muscle weakness, so clinician vigilance is essential. Table 1 summarizes the most common PFT abnormalities.

## 7. Bronchoalveolar Lavage

**Figure 7:**
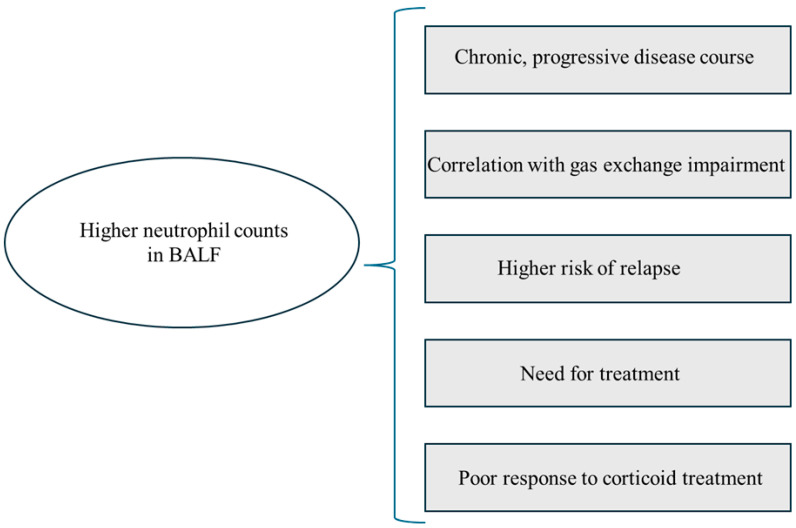
Graphic summary of the correlation between higher neutrophil counts and clinical presentation, pulmonary function test abnormalities, and management difficulties. BALF—bronchoalveolar lavage fluid.

Bronchoalveolar lavage (BAL), as a safe and relatively simple method, is one of the procedures used to diagnose ILDs. The analysis of BALF may possess prognostic significance and be utilized to evaluate disease activity. At diagnosis, one of the main features of pulmonary sarcoidosis is an increased lymphocyte T CD4 count and the CD4/CD8 ratio in the BALF [21,101]. Along with the radiographic stage of sarcoidosis, an increase in CD8 and a decrease in CD4 cell count, as well as in CD4/CD8 ratio, and an increase in neutrophil count and percentage in BALF are documented [22,102,103]. High BALF neutrophil counts correlated with a worse clinical course, greater gas exchange impairment, and higher relapse risk of pulmonary sarcoidosis [102,103,104,105]. Drent et al. also showed that a higher number of polymorphonuclear neutrophils (PMNs) was associated with the need for treatment, poor response to corticoids, and consideration of lung transplantation due to pulmonary fibrosis [103]. An association between a greater percentage of BALF neutrophils and the need for steroid therapy was observed in patients with both advanced and earlier radiological stages in a study by Ziegenhagen et al. [106]. However, all of these studies included a small number of patients with stage IV sarcoidosis. Moreover, the cut-offs for higher neutrophil counts vary among authors: Drent et al. [103] assumed 0.2 × 10^4^ cells/mL^−1^, Feng et al. [104] and Ziegenhagen et al. [106] used >3%, while in studies by Aleksoniene et al. [102] and Kollert et al. [105], the differences in neutrophil counts were expressed in relative terms without a specific cut-off being defined. The correlation between higher neutrophil counts in BALF and the clinical course, PFT impairment, and management difficulties is summarized in Figure 7. The need for further studies regarding neutrophils in BAL and their role in the pathogenesis of fibrosis in fPS patients is highlighted.

Higher percentages of eosinophils in BALF have also been associated with increased severity of gas exchange impairment in sarcoid patients [105].

Higher frequencies of circulating monocytes CD 14hi CD16hi (inflammatory monocyte subset) at the time of sarcoidosis diagnosis were more common in patients with chronic progressive disease [107].

## 8. Histological Findings

As previously stated, the histological hallmark of the disease is well-formed sarcoid granuloma, typically situated along the bronchovascular bundles and lymphatics. The sarcoid granuloma comprises aggregations of macrophages, epithelioid cells, and multinucleated giant cells, surrounded by T lymphocytes, fibroblasts, dendritic cells, and, in some cases, isolated collections of B lymphocytes [108]. Sarcoid granulomas do not have any unique features that distinguish them from other granulomatous diseases, but some particular characteristics may indicate the diagnosis [51,108,109,110]. Varying degrees of cellularity and fibrosis in the granulomas have been observed in patients with active sarcoidosis [111]. Small areas of fibrosis around the granulomas have also been reported [1]. Scar tissue due to the previous granuloma may sometimes persist over time [110]. Interstitial pneumonitis may occur around granulomas and is usually mild [27,112,113].

**Figure 8:**
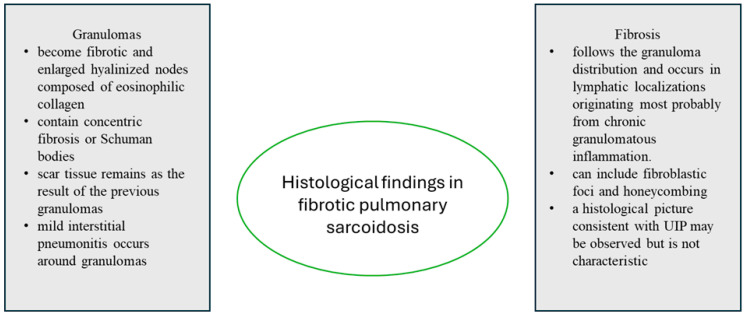
Summary of histological findings in fibrotic pulmonary sarcoidosis. UIP—usual interstitial pneumonia.

In sarcoidosis, pulmonary fibrosis generally follows the granuloma distribution and occurs in lymphatic localizations, originating most probably from chronic granulomatous inflammation [91,95,113]. In end-stage sarcoidosis, granulomas may become fibrotic and enlarged, replaced by hyalinized nodules composed of eosinophilic collagen [114]. Concentric fibrosis or concentric lamellar calcifications (Schumann bodies) are typical of fibrotic sarcoidosis [115]. Figure 8 summarizes the abovementioned information about granulomas and fibrosis in fPS.

Unfortunately, there are few studies analyzing the histopathological patterns in fPS [112,113,116]. In most cases, even in advanced pulmonary fibrosis, features of granulomatous inflammation were present, whereas in others, only features of advanced interstitial fibrosis were found. Features of the UIP pattern, such as fibroblastic foci and honeycombing, may be present in patients with end-stage pulmonary sarcoidosis [117]. In IPF, fibroblastic foci tend to be larger and more prevalent than those seen in fPS. Kamp et al. performed a molecular comparison of fibroblastic foci from IPF and sarcoidosis patients and showed their molecular similarity in both diseases. It may suggest that the pathogenic mechanisms of fibroblastic foci are independent of the underlying disease [118]. On the other hand, it is unclear if the UIP pattern in fPS patients undergoing lung transplantation could be a histological sign of lung injury in end-stage sarcoidosis or the coexistence of two interstitial lung diseases. Zhang et al. described lung explant findings from twelve patients with sarcoidosis. The authors found a typical picture of end-stage pulmonary sarcoidosis in eight of the twelve patients, characterized by the presence of well-formed granulomas with associated fibrosis, with a distinct lymphangitic distribution. In contrast, two sarcoidosis patients had a histological picture consistent with UIP, in one case with associated well-formed non-necrotizing granulomas in a lymphangitic distribution. The authors suggested that some patients with an established diagnosis of sarcoidosis might develop another fibrotic ILD, and in some patients, granulomas could resolve before transplantation. They emphasized that the most important feature in differentiating fPS from UIP/IPF is the typical distribution of pulmonary fibrosis in these diseases and the presence or absence of granulomas [119]. Shigemitsu et al. described the histopathology of explanted lungs of seven patients with sarcoidosis and found evidence of moderate-to-severe chronic interstitial pneumonitis in four of them, of whom two presented with the UIP pattern. The authors found the coexistence of two interstitial diseases unlikely, considering that both of these diseases are rare. They suggested that although the radiological and histological UIP pattern is a characteristic of IPF, it can also be observed in other ILDs with fibrosis [113]. An overlap of sarcoidosis and UIP/IPF features is widely considered [115,120,121]. Collins and colleagues in a very interesting study identified a large group of 25 patients with a diagnosis of both sarcoidosis and IPF, naming this syndrome combined sarcoidosis and idiopathic pulmonary fibrosis (CSIPF). They performed a comparative analysis with a control group of patients with lone-IPF and stage III and IV sarcoidosis. CSIPF patients were found to be similar to lone-IPF patients in terms of demographics, disease progression, and survival. The authors considered the coexistence of two independent disease entities (perhaps in individuals with a genetic predisposition), a syndrome with a different phenotype from IPF and sarcoidosis, or end-stage fibrosis in sarcoidosis patients. According to the authors, patients with a histopathological picture of UIP and features of sarcoidosis present remotely or concurrently should be considered as having IPF in terms of planned therapeutic management, including antifibrotic treatment or earlier listing of the patient for transplantation [121].

## 9. Complications

### 9.1. Infections

An increased risk of bacterial and fungal infection in patients with fPS is associated with both immunosuppressive or anti-TNF-α treatment and structural lung abnormalities such as bronchiectasis or fibrocystic areas [52,55,56].

**Figure 9:**
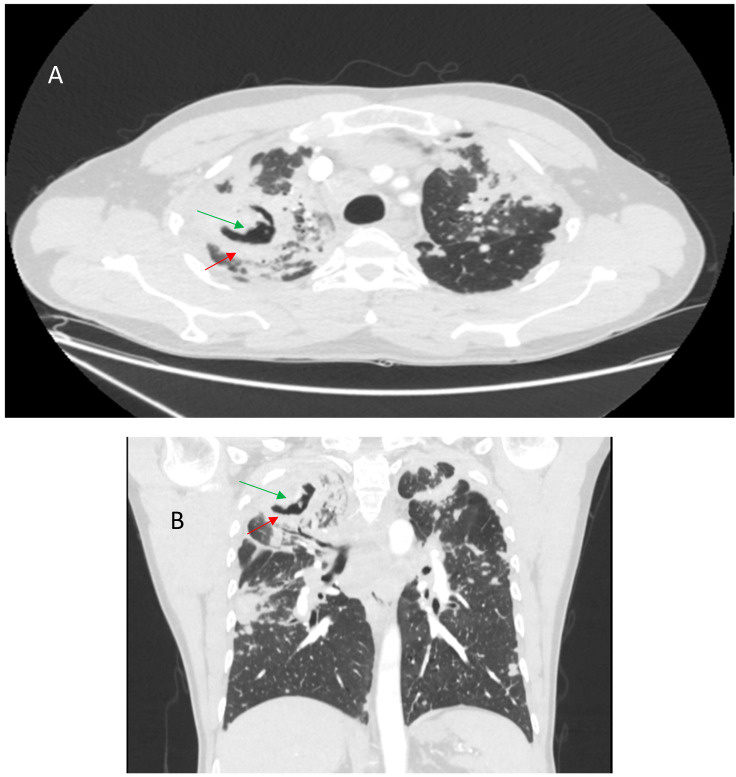
Axial CT scan (**A**) and coronal reconstruction (**B**) showing thick-walled cavity (red arrows) with mycetoma (green arrows) in the right upper lung lobe.

One of the most common infections that complicate fPS is CPA. Its frequency is variable. In the 2011 study by Nardi et al., CPA was observed in 11.3% of cases during a mean follow-up of 7.1 ± 4.8 years [52]. On the other hand, in a 2011 study by Pena et al., the frequency of CPA was 2% [55]. The most common phenotypes of CPA in patients with sarcoidosis are chronic cavitary pulmonary aspergillosis (CCPA) and aspergilloma. However, subacute invasive aspergillosis and overlaps between CCPA, aspergilloma, and chronic fibrosing pulmonary aspergillosis can be observed. Some phenotypes cannot be classified due to confusing radiological lesions [56]. The diagnostic criteria for CPA in patients with end-stage sarcoidosis presenting with cavities in the lungs include the occurrence of a fungal ball and/or serum Aspergillus IgG antibodies. Histological examination showing hyphae in biopsied cavities or cultures of Aspergillus spp. and/or PCR Aspergillus assays may support the diagnosis [122]. The most common symptoms (approximately 35–45%) of CPA-complicated sarcoidosis are hemoptysis, weight loss, and fatigue [56]. CPA is associated with poor prognosis and a high mortality rate [55]. However, mortality is usually related to advanced pulmonary sarcoidosis [56]. Management of CPA includes oral antifungal therapy and reduction of immunosuppressive drug doses when possible. Surgical treatment of CPA appears to be challenging given the advanced fibrocystic lesions on imaging [55,56]. Bronchial artery occlusion remains a solution for patients with massive hemoptysis [123]. Bacterial and mycobacterial coinfection is not a rare complication in sarcoid patients with CPA. In a study of patients with fPS and CPA, Uzunhan et al. described bacterial coinfection in 29% of patients and mycobacterial coinfection in 4.6%. Isolated pathogens included *Pseudomonas aeruginosa*, *Staphylococcus aureus*, *Enterobacter cloacae*, *Klebsiella pneumoniae*, *Streptococcus pneumoniae*, *Mycobacterium xenopii*, and *Mycobacterium avium* [56]. The possible picture of CPA in fPS is shown in Figure 9.

### 9.2. Pulmonary Hypertension and Pulmonary Embolism

PH is a common finding in sarcoid patients with a variable frequency of up to 50% and as high as 75% in patients awaiting lung transplantation. The risk of developing PH in sarcoidosis is higher in patients with extensive interstitial infiltrates and pulmonary fibrosis, with a predicted vital capacity of less than 60% and a predicted TLCO impairment of 60% [53,54,97]. However, it also occurs in earlier stages of the disease, highlighting the complex pathophysiology of PH in sarcoidosis [97,124]. Sarcoidosis-associated PH is classified as World Health Organization (WHO) class 5 PH with unclear and/or multifactorial mechanisms [125]. Direct compression of the pulmonary arteries, fibrotic destruction of the pulmonary vasculature due to parenchymal fibrosis, granulomatous changes of the pulmonary vasculature, hypoxia, and pulmonary vasculopathy are some of its possible causes [97,126]. As with other ILDs, patients with sarcoidosis are at an increased risk of pulmonary embolism and deep vein thrombosis, which may imply a higher risk for chronic thromboembolic PH [127,128,129].

### 9.3. Acute Exacerbations

Acute exacerbation of IPF is defined as clinically significant respiratory deterioration that develops within typically less than 1 month, accompanied by new radiological abnormalities on HRCT such as diffuse, bilateral ground-glass opacities with or without consolidation, and in the absence of other obvious clinical causes such as fluid overload, left heart failure, or pulmonary embolism [130]. Clinical deterioration may also occur in fPS. Unfortunately, there is no such definition for sarcoidosis, which makes it difficult to recognize and assess the frequency of exacerbations. However, acute exacerbations are not supposed to be rare in patients with fPS [131,132]. Some authors define them as worsening of pulmonary symptoms not explained by another cause, combined with a decline in spirometry (10% decrease in FVC and/or forced expiratory volume in 1 s (FEV1) from the previous baseline) [131]. Baughman and Lower studied the prevalence of acute worsening events, defined as episodes treated with either antibiotics and/or increased corticosteroid doses, in 129 patients with fPS and found their frequency ranged from 0 to 8 in the previous year. Risk factors for exacerbations were the presence of bronchiectasis on a chest CT scan and anti-TNFα treatment, reflecting more severe lung disease or increased immunosuppression [132]. Gazengel and colleagues investigated the causes of hospitalization and predictors of mortality in patients with sarcoidosis and showed that pulmonary exacerbations of sarcoidosis, which occurred in 11% of subjects, were the second most common reason for hospitalization after infections noted in 21% of patients. The presence of comorbidities and the need for oxygen therapy during hospitalization were associated with worse transplant-free survival [133]. Acute exacerbations appear to be a problem in patients diagnosed with sarcoidosis and UIP. Tachibana et al. reported a patient with both diseases who died of acute respiratory failure 3 years after the diagnosis of sarcoidosis. Features of UIP and superimposed diffuse alveolar damage (DAD) were seen on histopathological examination at autopsy. The authors speculated that an acute exacerbation developed in the course of UIP [115]. The lack of a determined definition of acute exacerbation of sarcoidosis hampers the management of sarcoidosis exacerbations. McKinzie et al. considered low-dose (20 mg daily) corticosteroid application sufficient action. However, this study included only patients treated chronically with 0–10 mg of prednisone daily, and the authors discussed patients with potentially more severe disease outcomes who were included in other studies. Baugman and Lower suggested the use of antibiotics, but the acute exacerbations in this study were likely related to infection rather than other causes [132].

## 10. Management

The management of fPS is challenging for the clinician due to the variable presentation and course of the disease, its complications, and the lack of evidence-based recommendations. Therapeutic decisions often depend on the clinical experience of a particular center and are based on expert opinion. In the absence of well-designed and conducted randomized, placebo-controlled clinical trials on the treatment of patients with fPS, there are no clear recommendations in the current European Respiratory Society (ERS), British Thoracic Society (BTS), or Delphi consensus guidelines [70,134,135]. Despite significant progress in recent years regarding the pathogenesis and predictive factors for the course of sarcoidosis, it is still unclear why a proportion of sarcoidosis patients develop pulmonary fibrosis despite adequate immunosuppressive treatment. There are also different phenotypes of fPS with diverse courses and prognoses [85,91,93]. Therefore, treatment should be individualized, based on a case-by-case scenario and multidisciplinary discussion. Considering the potential benefits and taking into account the toxicity of the treatment used, not every patient with fPS will require anti-inflammatory treatment. Therefore, numerous factors indicating disease activity or progression should be considered when planning treatment.

The use of anti-inflammatory medications in fPS is commonly discussed [91,95,136,137]. In cases of granulomatous inflammation accompanying pulmonary fibrosis, anti-inflammatory treatment should be considered, and the goals of this therapy should be clearly defined: prevention of progression, improvement/stabilization of lung function, and improvement of quality of life. The drugs used in the first line of treatment are corticosteroids at an initial dose recommended by the ERS and BTS, equivalent to 20–40 mg of prednisone [70,134]. Once the desired effect has been achieved within a few weeks, the corticosteroid dose is gradually reduced to a maintenance dose, usually 5–10 mg daily. Nevertheless, although the short-term efficacy of glucocorticoids has been shown, there are no controlled trials of the efficacy of glucocorticoid therapy in preventing lung function deterioration in advanced pulmonary sarcoidosis [134,138].

In case of disease progression, unacceptable side effects of steroids, the presence of serious comorbidities likely to be adversely affected by steroid therapy, or lack of patient agreement to the use of steroids, second-line treatment may be considered. The ERS guidelines suggest that methotrexate should be the preferred second-line treatment; however, alternative, glucocorticoid-sparing agents (azathioprine, leflunomide, mycophenolate mofetil) are also effective [134,139,140,141,142,143,144,145,146,147,148]. In the context of fPS, the use of methotrexate may pose some challenges, raising concerns about pulmonary toxicity. Methotrexate-induced pneumonitis is a rare complication in patients with sarcoidosis. In a large randomized controlled trial of methotrexate in cardiac sarcoidosis, the incidence of acute pneumonitis was 0.2% [149]. Anti-TNF medications are the third-line treatment, with infliximab as preferred [134,150,151,152]. Roflumilast, a phosphodiesterase-4 (PDE-4) inhibitor, which is used in the treatment of chronic obstructive pulmonary disease (COPD) with frequent exacerbations, can suppress oxidative stress and inflammation. It also has immunosuppressive properties. Baughman et al. in a small trial of 38 fPS patients showed that the use of roflumilast was associated with fewer exacerbations, improved quality of life, and improved FEV1 at the subsequent visit [153]. To establish the benefit of roflumilast treatment in fPS, further studies in a much larger group of patients are needed.

Recent studies have shown beneficial antifibrotic effects of the two drugs nintedanib and pirfenidone in the treatment of patients with IPF, and nintedanib in the treatment of patients with interstitial lung disease with progressive fibrosis [92,154,155,156]. However, no consensus has been reached on whether anti-fibrotic agents should be added or used in monotherapy as initial treatment in fPS patients. Nintedanib and pirfenidone combine anti-inflammatory and antifibrotic effects in their mechanism, which seems intriguing considering the complicated etiology of sarcoidosis.

Pirfenidone can decrease the production of proinflammatory cytokines, e.g., IL-1β, IL-6, and TNF-α [157,158]. Stroma cell-derived factor (SCDF/CXCL12), responsible for fibrocyte trafficking to the lung, can also be alleviated by pirfenidone [157,159]. Anti-fibrotic action is associated with suppressing the TGF-β-induced fibroblast proliferation and collagen synthesis and downregulating platelet-derived growth factor (PDGF) and basic fibroblast growth factor (bFGF). IL-4 and IL-13 are also decreased by pirfenidone treatment [158,160]. Pirfenidone is recommended for patients with IPF [161]. The safety and efficacy of treatment with pirfenidone compared to placebo in patients with ILD other than IPF was to be assessed in a prospective, randomized (1:1), placebo-controlled clinical RELIEF trial, but the study was prematurely terminated because of slow recruitment. However, a significantly lower decline in FVC % predicted in the pirfenidone group was observed. Patients with sarcoidosis were not included in this trial [162]. Pirfenidone was also investigated in a double-blind, randomized, placebo-controlled, phase 2 trial, including patients with progressive fibrosing unclassifiable ILD, which indicated that they could benefit from pirfenidone treatment [163]. The multi-center, double-blind, placebo-controlled, feasibility trial of pirfenidone for patients with significant fPS (Pirfenidone for Fibrotic Sarcoidosis [PirFS]) was to evaluate pirfenidone efficacy in this group of patients. Due to difficulties caused by international regulations excluding sites, slow patient recruitment, and the COVID-19 pandemic, it was terminated prematurely and was underpowered to assess the efficacy of pirfenidone in fPS [87].

Nintedanib is a small-molecule tyrosine kinase inhibitor acting via PDGF receptor-α and -β, FGF receptor-1–3, and vascular endothelial growth factor (VEGF) receptor-1–3 [164]. Referring to the mechanisms mentioned above that may play a role in the pathogenesis of fibrosing sarcoidosis, nintedanib inhibits the release of mediators, including IL-2 (and its production), IL-4, IL-5, IL-10, IL-12p70, IL-13, and IFN-γ, by human peripheral blood mononuclear cells or T-cells [164]. It alters macrophage polarization into an M2 phenotype. [165] Nintedanib has been approved for IPF treatment after positive results from the INPULSIS study. Data from the INBUILD study, which evaluated the efficacy and safety of nintedanib in treating patients with PF-ILD other than IPF, showed a statistically significant reduction in disease progression, assessed as the decline of FVC per year. Based on this trial, nintedanib has reached a conditional recommendation in patients with progressive pulmonary fibrosis (PPF). However, only 12/663 patients with sarcoidosis were included, and therefore, it is difficult to draw clear conclusions on the effect of nintedanib treatment in this group of patients [92]. Theoretically, patients with fPS presenting UIP-like pattern on histology or HRCT or with progressive disease despite anti-inflammatory treatment could benefit similarly to IPF or other PPF patients. The recently published guidelines of the Austrian Society for Rheumatology and the Austrian Society for Pulmonology for the diagnosis and treatment of sarcoidosis recommend antifibrotic treatment with nintedanib for progressive fibrosis associated with sarcoidosis [166]. Undoubtedly, further studies are needed on the efficacy of antifibrotic treatment dedicated to patients with fibrotic pulmonary sarcoidosis.

Some positive reports of pulmonary rehabilitation programs (PR) in patients with advanced pulmonary sarcoidosis include an increase in 6MWD, oxygenation, muscle strength, improvement in the Saint George’s Respiratory Questionnaire, reduced dyspnea, fatigue, anxiety, and depression [167,168]. Wallaert et al., in an open-label multicenter randomized controlled trial for patients with stage IV sarcoidosis evaluating the efficacy of a 2-month PR program, found significant improvement in dyspnea, exercise tolerance, and quality of life compared with baseline. However, none of the daily life physical activity measures (including the number of steps per day, total daily energy expenditure, and total energy expenditure above 2.5 metabolic equivalents [METs]) improved immediately post-PR and in the long-term after PR. No long-term improvement was shown in the mean values for FVC and TLCO (% predicted) [169].

Data on an increased infection risk in sarcoidosis patients considering auto/immunological pathogenesis, and therapy with corticoids, immunosuppressive, and anti-TNF-α agents may suggest the need for prophylaxis. Vaccination should be considered in conjunction with guidelines for chronic lung diseases, regarding immunosuppressive treatment and the type of vaccine. The recommendations for vaccinations in immunosuppressed patients may be considered. Vaccination strategies should be executed at the time of stable disease and evaluated during routine follow-up [170].

### Lung Transplantation

Lung transplantation serves as a last resort for patients with fPS suffering from respiratory failure or PH. Advanced pulmonary sarcoidosis accounts for 2.5–3.5% of cases referred for lung transplant assessment [171,172]. The criteria for listing contain severe impairment in lung function, insufficient reaction to medical treatment, and marked physical activity limitation with a New York Heart Association functional (NYHA) class III or IV [173]. Prioritization depends on the patient’s mean pulmonary artery pressure (mPAP) because PH accelerates clinical decline [174]. Arcasou et al. evaluated patients with sarcoidosis in the University of Pennsylvania Lung Transplant Program and found a mortality rate on the waitlist close to 50% of listed patients with a median survival time of 2 years. Hypoxemia, PH, decreased cardiac index, and elevated right atrial pressure (RAP) have been associated with a significant risk of death [173]. In contrast, in a recent study, Gangemi et al. found an 18.2% (6 of 27 subjects) mortality among sarcoidosis patients listed for lung transplant, compared to a 12.3% mortality among IPF patients and an 8.7% mortality among COPD patients. However, the reasons for deaths were not determined in all cases. No differences concerning prednisone dose and immunosuppressant use were noted between patients who survived to transplantation compared to those who died on the waitlist. Importantly, TLCO and Composite Physiologic Index (CPI) were significantly higher in the transplanted population compared to those who died. Eleven of the patients had a secondary listing diagnosis of PH [171]. Sarcoid lung recipients present a median post-transplant survival rate of 70 months, similar to other indications [172]. Taking all this into account, it seems reasonable to consider transplantation in all cases of end-stage sarcoidosis, remembering waitlist time and the reasons for not being listed, including serious comorbidity, extremes of weight, severe debility, and peripheral mycetomas connected with extensive pleural thickening [173].

**Figure 10:**
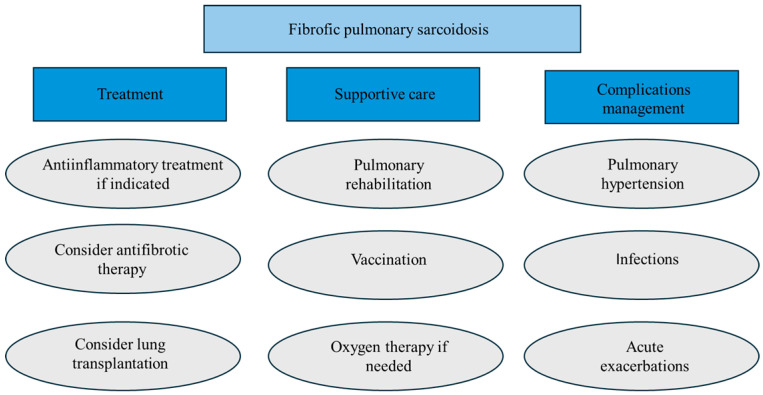
Summary of fibrotic pulmonary sarcoidosis management.

Figure 10 briefly summarizes the holistic management of fPS.

## 11. Prognosis and Mortality

Increased sarcoidosis-related mortality has been observed [175,176,177]. Pulmonary fibrosis is one of the major causes of mortality in sarcoidosis [178]. In an interesting study conducted by Nardi et al. in patients with stage IV sarcoidosis, mortality was related to respiratory causes in 75% of cases, and survival was decreased compared to the general population [52]. Cardiac involvement is another leading cause of mortality in sarcoidosis [179]. Recent innovations in echocardiographic imaging have led to speckle-tracking echocardiography (STE) development. A recent systematic review and meta-analysis have demonstrated that STE analysis may allow the early detection of myocardial involvement in sarcoidosis patients [180].

Wells et al., in a study on IPF patients, have presented a new index- CPI- that reflects the extent of interstitial disease more precisely than individual lung function indices and provides prognostic data. The formula is: the extent of disease on CT = 91.0 − (0.65 × percent predicted TLCO) − (0.53 × percent predicted FVC) − (0.34 x percent predicted FEV1) [181]. The CPI has also been shown to have predictive value in patients with sarcoidosis [65,182]. In addition, more than 20% of pulmonary fibrosis on HRCT and PH are associated with worse survival [63,65,66,126,182].

## 12. Conclusions and Future Directions

fPS represents a complex and potentially debilitating phenotype associated with reduced quality of life, impaired lung function, and increased mortality. It remains an open question of how to assess whether a patient has an active inflammatory process that would suggest a benefit from anti-inflammatory treatment. The optimal management of fPS requires a multidisciplinary team approach. Clinical symptoms, serum biomarkers, imaging studies such as HRCT, and in some cases, PET/CT, results of PFTs, other organ involvement, comorbidities, and complications should be considered to guide the intensity and duration of therapy. In mild or stable cases, observation or low-dose immunosuppression may be sufficient. Conversely, patients with extensive fibrosis, UIP-like patterns, or high-risk HRCT features (e.g., basal subpleural honeycombing) may warrant more aggressive strategies, including consideration of antifibrotic agents, advanced evaluation for lung transplantation, or enrollment in clinical trials. Currently, recruiting and ongoing clinical trials are listed in Table 2.

## Figures and Tables

**Table 1:** The most common pulmonary function test abnormalities in patients with fibrotic pulmonary sarcoidosis. TLCO—lung transfer factor for carbon monoxide, PH—pulmonary hypertension, HRCT—high-resolution computed tomography, 6-MWT—six-minute walk test, DSP—distance-saturation product.

Test	Expectation in Fibrotic Sarcoidosis	Comments
TLCO	Decreased	-the most common abnormality in patients with fPS; -if lower, increased risk of clinical worsening and death; -higher impairment requires PH searching.
Plethysmography	restriction/	-the most common plethysmography defect in patients with fPS; -associated with a honeycomb pattern on HRCT; -increased mortality.
	obturation/	-caused by granuloma formation and inflammation in airways or airway distortions, bullous changes, and peribronchial or peribronchiolar fibrosis; -no association with mortality confirmed.
	mixed ventilatory defect	-the most common defect according to some authors; -associated with longer disease duration; -often in smokers.
6-MWT	shorter walk distance, greater desaturation, and higher heart rate	-desaturation found as a more valuable prognostic feature than distance; -greater degrees of desaturation correlate with PH; -DSP for consideration.

**Table 2:** Clinical trials in sarcoidosis are recruiting/ongoing. CHIT1—chitotriosidase 1; GM-CSF—granulocyte-macrophage colony-stimulating factor; PDE4B—phosphodiesterase 4B.

Clinical Trial	Drug	Mechanism of Action	
Xentria, phase 1b/2 study	XTMAB-16	monoclonal antibody against TNF-α	
KITE, phase II clinical trial	OATD-01	inhibitor of CHIT1	
RESOLVE-Lung, phase II clinical trial	Namilumab	human monoclonal antibody against GM-CSF	
EFZO-FIT, phase III clinical trial	Efzofitimod	immunomodulator binding the neuropilin 2 receptor protein	
FIBRONEER-ILD, phase III clinical trial	Nerandomilast	preferential inhibitor of PDE4B	

**Future directions:**			
Multidisciplinary team approach. Multicenter, international clinical trials of genetic background, biomarkers, and environmental factors influencing sarcoidosis. Applying novel technologies like NGS, WES, AI in integrating sarcoidosis phenotyping. Randomized clinical trial of anti-inflammatory and antifibrotic drugs in fPS patients.			

## Data Availability

Not applicable.

## Abbreviations

The following abbreviations are used in this manuscript:

fPS	Fibrotic pulmonary sarcoidosis
IL	Interleukin
Th	T helper
M1	A proinflammatory subtype of macrophages
M2	Alternatively activated macrophages
TNF-α	Tumor necrosis factor-α
TGF-β	Transforming growth factor-β
IFN-γ	Interferon-γ
CCL 18	CC chemokine ligand 18
CT	Computed tomography
HRCT	High-resolution computed tomography
PFT	Pulmonary function test
TLCO	Lung transfer factor for carbon monoxide
6-MWT	Six-minute walk test
FEV1	forced expiratory volume in 1 s
FVC	Forced vital capacity
FAS	Fatigue Assessment Scale
PH	Pulmonary hypertension
BAL	Bronchoalveolar lavage
BALF	Bronchoalveolar lavage fluid
CPI	Composite Physiologic Index
DSP	Distance-saturation product
ILD	Interstitial lung disease
PF-ILD	Progressive fibrosis- interstitial lung disease
UIP	Usual interstitial pneumonia
IPF	Idiopathic pulmonary fibrosis
ERS	European Respiratory Society
BTS	British Thoracic Society
CTD	Connective tissue disease
COPD	Chronic obstructive pulmonary disease
DAD	Diffuse alveolar damage
WHO	World Health Organisation
NYHA	New York Heart Association
CPA	Chronic Pulmonary Aspergillosis
CSIPF	combined sarcoidosis and idiopathic pulmonary fibrosis
PMNs	Polymorphonuclear neutrophils
PDGF	Platelet-derived growth factor
bFGF	Basic fibroblast growth factor
VEGF	Vascular endothelial growth factor
PR	Pulmonary Rehabilitation
NGS	next-generation sequencing
WES	Whole-exome sequencing
AI	Artificial Intelligence
